# Proteoglycan binding as proatherogenic function metric of apoB-containing lipoproteins and chronic kidney graft failure

**DOI:** 10.1016/j.jlr.2021.100083

**Published:** 2021-04-30

**Authors:** Hannah L.M. Steffen, Josephine L.C. Anderson, Margot L. Poot, Yu Lei, Margery A. Connelly, Stephan J.L. Bakker, Katariina Öörni, Uwe J.F. Tietge

**Affiliations:** 1 Department of Pediatrics, University of Groningen, University Medical Center Groningen, Groningen, The Netherlands; 2 Division of Clinical Chemistry, Department of Laboratory Medicine, Karolinska Institutet, Stockholm, Sweden; 3 Laboratory Corporation of America Holdings (LabCorp), Morrisville, NC, USA; 4 Department of Nephrology, University of Groningen, University Medical Center Groningen, Groningen, the Netherlands; 5 Atherosclerosis Research Laboratory, Wihuri Research Institute, Helsinki, Finland; 6 Molecular and Integrative Bioscience Research Programme, Faculty of Biological and Environmental Sciences, University of Helsinki, Helsinki, Finland; 7 Clinical Chemistry, Karolinska University Laboratory, Karolinska University Hospital, Stockholm, Sweden

**Keywords:** kidney transplantation, chronic graft failure, transplant vasculopathy, proteoglycans, cholesterol, atherosclerosis, LDL, prospective, lipoprotein-proteoglycan binding susceptibility, cardiovascular mortality, eGFR, estimated glomerular filtration rate, GF, graft failure, HR, hazard ratio, LPBS, lipoprotein-proteoglycan binding susceptibility, RTR, renal transplant recipient, sPLA_2_-IIA, group IIA phospholipase A_2_, TC, total cholesterol, TV, transplant vasculopathy

## Abstract

Lipoprotein-proteoglycan binding is an early key event in atherosclerotic lesion formation and thus conceivably could play a major role in vasculopathy-driven chronic graft failure and cardiovascular mortality in renal transplant recipients. The present study investigated whether lipoprotein-proteoglycan binding susceptibility (LPBS) of apoB-containing lipoproteins and levels of the classical atherosclerosis biomarker LDL-C were associated with cardiovascular mortality (n = 130) and graft failure (n = 73) in 589 renal transplant recipients who were followed up from at least 1 year after transplantation for 9.5 years. At baseline, LPBS was significantly higher in patients who subsequently developed graft failure than in those with a surviving graft (1.68 ± 0.93 vs. 1.46 ± 0.49 nmol/mmol, *P* = 0.001). Cox regression analysis showed an association between LPBS and chronic graft failure in an age- and sex-adjusted model (hazard ratio: 1.45; 95% CI, 1.14–1.85; *P* = 0.002), but no association was observed with cardiovascular mortality. LDL-C levels were not associated with graft failure or cardiovascular mortality. This study shows that measurement of cholesterol retention outperformed the traditionally used quantitative parameter of LDL-C levels in predicting graft failure, suggesting a higher relevance of proatherogenic function than the quantity of apoB-containing lipoproteins in chronic kidney graft failure.

Atherosclerosis negatively impacts the prognosis of renal transplant recipients (RTRs) in two ways, (i) in the form of pre-existing, mostly complex atherosclerotic lesions, which are the underlying pathology for CVD and (ii) as de novo atherosclerotic lesion formation in the graft, known as transplant vasculopathy (TV), the single major cause for chronic graft failure (GF) (1, 2, 3). Patients receiving kidney grafts often have a long-standing history of end-stage renal disease with dialysis treatment, which is per se associated with a 30- to 40-fold increase in age-adjusted CVD mortality (4). After transplantation, CVD risk is still 4- to 6-fold higher and is the leading cause of death in RTRs (4). A decrease in renal function over time further contributes to the increased risk. The substrate for this chronic graft functional decline is TV, an atherosclerotic process in the vasculature of the transplanted organ, affecting 50% of allografts after 5 years and 90% after 10 years (2, 3). Importantly, in RTRs, classical CVD risk factors, such as levels of LDL-C or HDL-C, fail to serve as predictive biomarkers either for CVD events or for de novo atherosclerosis leading to TV-mediated chronic GF (1, 3, 5). Thus, the identification of predictive biomarkers represents an unmet clinical need in this patient population. Recent studies indicated that assays capturing the functional properties of HDL lipoproteins might provide clinical information beyond cholesterol levels within these, exemplified by HDL cholesterol efflux capacity being able to predict CVD in the general population (6, 7) and chronic GF in RTRs (8), independent of HDL-C levels. However, throughout the atherogenic process, especially LDL particles play a central and pivotal role (9). Specifically, binding of LDL particles to proteoglycans in the vessel wall is an early key event in the initiation of atherosclerotic lesions, as summarized in the now widely accepted response-to-retention hypothesis of atherogenesis (10). However, thus far, the concept that measures of LDL functionality can be used as predictive biomarkers has not been widely explored. Especially, the susceptibility of LDL to bind to vascular proteoglycans appears promising in this respect. Therefore, in the present work, we investigated, whether in comparison to LDL-C levels, the lipoprotein-proteoglycan binding susceptibility (LPBS) of apoB-containing lipoproteins is prospectively associated with the two clinically relevant atherosclerosis-related outcomes in RTRs, CVD mortality on the one hand and chronic GF on the other.

## Materials and methods

### Study design and study population

For this prospective study, an established and well-characterized patient cohort of adult RTR (TransplantLines) was used (8, 11). Patients were recruited at the outpatient clinic of the University Medical Center Groningen between August 2001 and July 2003. To lessen the potential confounding effect of early immune-mediated rejection, patients were required to have, at inclusion, a functioning graft for at least 1 year after transplantation. Exclusion criteria were overt congestive heart failure, endocrine abnormalities other than diabetes mellitus, malignancies other than cured skin cancer, and suspected acute infection. 606 of 847 eligible patients volunteered to participate and were included in the cohort (72% consent rate). Nonparticipants were compared with participants with regard to age, sex, BMI, plasma creatinine, creatinine clearance, and proteinuria, and no significant differences were found (8, 11). Written informed consent was obtained from all participants. The institutional review board approved the study protocol (METc2001/039), which complied with the Declaration of Helsinki. The study is registered at ClinicalTrials.gov with identifier NCT03272854 under the name “The TransplantLines Insulin Resistance and Inflammation Biobank and Cohort Study”.

### Outcome measures and end points of the study

The main outcome measure of this study is LPBS. Primary end points are (i) death-censored GF, defined as return to dialysis therapy or retransplantation and (ii) cardiovascular mortality, defined as deaths with the principal cause of death being CV in nature, using codes 410–447 of the International Classification of Diseases, ninth revision (12). If the status of a patient was unknown, general practitioners or referring nephrologists were contacted. Subjects’ status regarding survival and GF was recorded until April 2012. Causes of death were available until May 2009. One subject was lost to follow-up.

### Baseline measurements and definitions

Transplant characteristics, such as subject demographics, previous history, as well as date and type of transplantation, were extracted from the Groningen Renal Transplant Database. Smoking status and CVD history (considered positive if participants previously had a myocardial infarction, transient ischemic attack, or cerebrovascular accident) were obtained using a self-report questionnaire at inclusion. Medical records were consulted for information on use of medication. Standard immunosuppressive regimen changed over the years: from 1968 to 1989, prednisolone and azathioprine (100 mg/day); from January 1989 to February 1993, cyclosporine standard formulation (10 mg/kg; trough levels of 175–200 μg/l in the first 3 months, 150 μg/l between 3 and 12 months after transplantation, and 100 μg/l thereafter) and prednisolone (initially 20 mg/day, rapidly tapered to 10 mg/day); from March 1993 to May 1997, cyclosporine microemulsion (10 mg/kg, trough levels as before) and prednisolone; and from May 1997 onward, mycophenolate mofetil (2 g/day) was added (13).

Blood samples were drawn at time of inclusion after an 8–12 h overnight fasting period. No specific antioxidant was added at the time of blood draw; however, all samples contained EDTA, a known antioxidant, and were handled in an identical fashion. All standard laboratory measures, including hemoglobin A1C (HbA1c), were performed at time of inclusion. Concentrations of total cholesterol (TC) and HDL-C were determined using the cholesterol oxidase-phenol aminophenazone method. Concentration of LDL-C was calculated using the Martin Hopkins equation (14). apoA-I, apoB, and lipoprotein (a) [LP(a)] were determined by nephelometry with reagents for Dade Behring nephelometer systems (BN II, Siemens, Marburg, Germany). The glycerol-3-phosphate oxidase-phenol aminophenazone method was used to measure plasma triglycerides. Levels of plasma high-sensitive C-reactive protein were assessed by ELISA (8). Plasma glucose levels were determined using the glucose-oxidase method, and plasma insulin was measured using an AxSYM autoanalyzer. Levels of glycosylated HbA1c were assessed by high-performance liquid chromatography (8). homeostasis model assessment of insulin resistance was used to determine insulin resistance by multiplying glucose (mmol/l) with insulin (μU/ml) and dividing the result by 22.5 (15). Creatinine concentrations in plasma and urine were determined using a modified version of the Jaffé method. The estimated glomerular filtration rate (eGFR) was calculated using the Chronic Kidney Disease Epidemiology Collaboration formula (16); all values included in the analyses were obtained at time point of inclusion. Creatinine clearance was calculated from 24-h urinary creatinine excretion and plasma creatinine. Total urinary protein concentration was analyzed using the Biuret reaction.

Proteinuria was defined as urinary protein excretion ≥0.5 g per 24 h. The diagnosis of diabetes mellitus was made if antidiabetic medication was used or fasting plasma glucose concentration was ≥ 7.0 mmol/l or HbA1c was >6.5% (17). The BMI was calculated by dividing the weight in kilograms by the height in meters squared. Blood pressure was measured automatically in 1 min intervals after a 6 min rest in the supine position (Omron M4; Omron Europe BV, The Netherlands), and the mean of three measurements was taken.

The LDL particle number and average size were determined in EDTA plasma (n = 158 participants) by ^1^H-NMR spectroscopy using a Vantera® NMR Clinical Analyzer as previously described (Labcorp, Raleigh, NC) (18). Plasma group IIA phospholipase A_2_ (sPLA_2_-IIA) was assessed in n = 40 participants with an ELISA (Cayman Chemical, Ann Arbor, MI) according to the manufacturer’s instructions. Conjugated dienes were determined in apoB-containing lipoproteins (n = 40 participants) by absorbance at 233 nm after lipid extraction with dichloromethane/methanol (2:1, v/v) (19) using precipitates generated by polyethylene glycol-6000 precipitation of apoB-containing lipoproteins as described (6). Values were normalized for LDL-C levels.

### Laboratory analysis of lipoprotein binding to proteoglycans

EDTA plasma samples of 589 participants were available for the laboratory analysis of this study. The plasma samples were collected at baseline, placed on ice, centrifuged at 4°C, immediately stored at −80°C, and left unthawed until analysis. The samples were stored from time of inclusion until 2019.

To determine the binding susceptibility of lipoproteins to proteoglycans, human aortic proteoglycans were isolated from the intima-media of atherosclerotic human aortas (20), and the glycosaminoglycan content of proteoglycans was quantified as overall marker of proteoglycans (21). Then, wells of polystyrene 96-well plates (Thermo Fisher Scientific) were coated with 100 μl of proteoglycans (50 μg/ml in PBS) by incubation at 4°C overnight. Wells were blocked with 1% bovine serum albumin in PBS for 1 h at 37°C. Wells without proteoglycan coating served as controls for unspecific binding. To measure lipoprotein binding to the immobilized proteoglycans, 1 μl of plasma (derived from RTRs at baseline) was added to the wells in a buffer containing 140 mmol/l NaCl, 2 mmol/l MgCl_2_, 5 mmol/l CaCl_2_, and 10 mmol/l MES, pH 5.5, and incubated for 1 h at 37°C. The wells were washed with 10 mmol/l MES-50 mmol/l NaCl, pH 5.5, and the amount of bound TC was determined using the Amplex Red cholesterol kit (Molecular Probes). Each sample was analyzed in duplicate and the nonspecific binding in a single well had been blocked by the blocking buffer. The nonspecific binding consistently accounts for about 5% of the binding to the PG-coated wells. The assay was performed over a duration of several weeks. The day-to-day variation of the measurement is <15% and to control for this, a control plasma sample is analyzed in each plate. However, no drift in the assay was noted. The variation between duplicate measurements carried out in separate microtiter well plates is 2.3%. To correct for interindividual differences in proatherogenic lipoproteins in each individual sample, the amount of bound TC was divided by the concentration of plasma LDL-C. Results are thus expressed as nmol bound TC/mmol plasma LDL-C. This measure gives a realistic reflection of the binding susceptibility of plasma proatherosclerotic lipoproteins to proteoglycans in the arterial vessel wall. More than 90% of TC that binds to lipoproteins from plasma has been shown to be associated with LDL particles (21, 22).

### Statistical analysis

Baseline characteristics of the study population were analyzed for gender-stratified tertiles of levels of bound TC/plasma LDL-C (low, medium, and high). Normally distributed continuous variables are depicted as the mean ± standard deviation, whereas continuous variables with a skewed distribution are given as the median [25th–75th percentile]. Categorical variables are summarized by absolute numbers (percentages).

Baseline characteristics were tested for differences among groups with low, medium, and high LPBS based on sex-stratified tertiles. Baseline characteristics for normally distributed continuous variables were tested for differences among groups with one-way ANOVA. The Kruskal-Wallis test was used to assess differences between groups for continuous variables with a skewed distribution. Group differences in categorical data were tested with Pearson chi-squared test.

LPBS of subjects who reached the respective end points was compared with values of subjects not reaching the end points of the study by independent samples *t* test. Similarly, the LPBS for males and females was computed using independent samples *t* test. Subsequently, all characteristics with a *P* < 0.10 across gender-stratified tertiles of LPBS were entered into a step-wise multivariable linear regression model with backward elimination (*P* < 0.05) to identify variables independently associated with LPBS.

Multivariable Cox regression was used to calculate hazard ratios (HRs) and 95% CI for the primary end points. Adjustment of potential confounders was used to assess the independent association of LPBS with the end points chronic GF and cardiovascular mortality. Potential confounders were determined as known risk factors of chronic GF and CVD in RTRs and included age, sex, eGFR, periods of acute rejection, number of human leukocyte antigen mismatches, primary renal disease, diabetes mellitus, BMI, dialysis time, type of transplantation, use of calcineurin inhibitors, use of proliferation inhibitors, use of statins, time between transplantation and baseline, and donor age. Validity of proportional hazard assumptions was tested using Schoenfeld residuals. Furthermore, subgroup analysis using interaction tests were performed in which HRs were determined across categories of baseline characteristics. For continuous variables, the median value was used as cutoff. For the end point chronic GF, the subject characteristics were sex (male vs. female), age (<52.1 vs. >52.1 years), use of statins (yes vs. no), eGFR (<46.7 and ≥46.7 ml/min per 1.73 m^2^), and period of acute rejection (yes vs. no).

To compare the relevance of the proposed novel functionality parameter with a traditional quantitative parameter, statistical analyses were repeated for plasma concentration of LDL-C at baseline and results were compared with those of LPBS.

Two-sided *P*-values <0.05 were considered to indicate statistical significance. All statistical analyses and visualization of data were conducted using STATA® Statistical Software, Release 15.1 (StataCorp, College Station, TX).

## Results

In this longitudinal follow-up study, the LPBS was measured in 589 RTRs. Individual LPBS values were expressed as the ratio between proteoglycan-bound cholesterol and plasma LDL-C levels (nmol/mmol). Baseline characteristics according to sex-stratified tertiles of LPBS are summarized in Table 1. The concentrations of TC, LDL-C, apoA-I (each *P* < 0.001), triglycerides (*P* = 0.004), apoB (*P* = 0.012), and the LDL-C/apoB ratio (*P* < 0.001) decreased significantly with increasing tertiles of LPBS. Systolic blood pressure (*P* = 0.04) and BMI (*P* = 0.015) showed a significant inverse association with binding susceptibility, but for other potential cardiovascular risk factors including age, history of cardiovascular events, diabetes mellitus, tobacco abuse, plasma triglycerides, and HDL-C, no relationship with LPBS was evident.

**Table 1:** Baseline characteristics according to sex-stratified tertiles of lipoprotein-proteoglycan binding susceptibility

	Tertiles of Lipoprotein-Proteoglycan Binding Susceptibility (Bound TC/LDL-C)			*P*
	First (n = 197)	Second (n = 196)	Third (n = 196)	
Outcome parameter				
Lp-PG binding susceptibility, nmol/mmol	1.04 (0.94, 1.01)	1.29 (1.21, 1.34)	1.72 (1.64, 1.88)	<0.001
Recipient demographics				
Age, years	54.1 (43.9, 60.9)	52.8 (42.3, 61.0)	50.7 (41.5, 59.8)	0.099
Male gender, n (%)	109 (55.1%)	108 (55.1%)	108 (55.1%)	1.00
Current smoking, n (%)	71 (35.9%)	82 (42.5%)	81 (41.3%)	0.36
Body composition				
BMI, kg/m^2^	26.8 ± 4.4	25.8 ± 4.2	25.6 ± 4.2	0.015
Waist circumference, men, cm	101.4 ± 11.8	99.0 ± 12.6	98.9 ± 12.8	0.23
Waist circumference, women, cm	95.0 (85.5, 104.5)	91.5 (81.0, 101.0)	93.5 (82.0, 104.0)	0.21
Waist-hip ratio, women	0.91 (0.85, 1.00)	0.90 (0.85, 0.97)	0.94 (0.85, 1.02)	0.068
Waist-hip ratio, men	1.02 (0.96, 1.07)	1.00 (0.95, 1.08)	1.02 (0.96, 1.08)	0.25
Lipids				
TC, mmol/l	5.89 ± 1.10	5.69 ± 1.07	5.28 ± 1.00	<0.001
LDL-C, mmol/l	3.72 ± 0.97	3.64 ± 0.94	3.29 ± 0.96	<0.001
HDL-C, mmol/l	1.12 ± 0.34	1.11 ± 0.30	1.05 ± 0.33	0.054
apoA-I, g/l	1.57 (1.39, 1.78)	1.59 (1.41,1.78)	1.45 (1.29,1.65)	<0.001
apoB, g/l	1.10 (0.96, 1.24)	1.06 (0.93, 1.23)	1.01 (0.90, 1.21)	0.012
Triglycerides, mmol/l	2.21 (1.57, 2.92)	1.87 (1.41, 2.40)	1.82 (1.29, 2.41)	0.004
LDL-C/apoB	3.52 ± 0.94	3.35 ± 0.56	3.08 ± 0.71	<0.001
CVD history				
Myocardial infarction, n (%)	14 (7.1%)	14 (7.2%)	20 (10.3%)	0.43
TIA/CVA, n (%)	14 (7.1%)	9 (4.6%)	9 (4.6%)	0.47
Systolic blood pressure, mmHg	152.5 (139.0, 167.0)	150.0 (137.0, 167.0)	149.0 (132.5, 163.0)	0.037
Medication				
Antihypertensive drugs, n (%)	177 (89.4%)	166 (84.7%)	171 (87.2%)	0.38
Calcineurin inhibitors, n (%)	151 (76.3%)	160 (81.6%)	151 (77.0%)	0.38
Proliferation inhibitors, n (%)	142 (71.7%)	140 (71.4%)	152 (77.6%)	0.30
Use of statins, n (%)	100 (50.5%)	105 (53.6%)	91 (46.4%)	0.37
Glucose homeostasis				
Glucose, mmol/l	4.65 (4.20, 5.14)	4.45 (4.08, 5.02)	4.52 (4.10, 4.88)	0.22
HbA1c, %	6.30 (5.90, 7.10)	6.40 (5.80, 6.90)	6.40 (5.80, 7.003)	0.44
HOMA-IR	2.31 (1.62, 3.45)	2.33 (1.64, 3.31)	2.29 (1.55, 3.80)	0.89
Diabetes mellitus, n (%)	34 (18.5%)	30 (16.5%)	30 (16.0%)	0.79
C-reactive protein, mg/l	2.08 (0.95, 4.76)	1.82 (0.68, 4.49)	2.32 (0.76, 5.43)	0.55
Donor demographics				
Age, years	37.0 (22.0, 49.0)	35.5 (23.5, 48.0)	41.0 (24.0, 52.0)	0.27
Male gender, n (%)	109 (55.3%)	110 (56.4%)	102 (52.3%)	0.70
Living kidney donor, n (%)	26 (13.1%)	25 (12.8%)	27 (13.8%)	0.96
Number of HLA mismatches	1.00 (0.00, 2.00)	1.00 (0.00, 2.00)	1.00 (0.00, 2.00)	0.64
(Pre)transplant history				
Acute rejection, n (%)	92 (46.5%)	90 (45.9%)	83 (42.3%)	0.67
Dialysis time, month	26.0 (13.0, 47.0)	30.0 (17.0, 48.0)	27.0 (12.0, 52.0)	0.35
Time between Tx and inclusion, month	80.5 (37.0, 149.0)	70.0 (28.5, 134.0)	67.0 (34.0, 117.0)	0.11
Renal allograft function				
eGFR, ml/min/1.73 m^2^	46.3 ± 15.2	47.4 ± 15.2	47.1 ± 16.9	0.78
Urinary protein excretion, g/24 h	0.20 (0.00, 0.50)	0.20 (0.00, 0.50)	0.30 (0.20, 0.60)	0.13
Proteinuria ≥0.5 g/24 h, n (%)	50 (25.3%)	54 (27.8%)	62 (31.6%)	0.37

CVA, cerebrovascular accident; eGFR, estimated glomerular filtration rate; HbA1c, hemoglobin A1c; HOMA-IR, Homeostatic Model Assessment for Insulin Resistance; HLA, human leukocyte antigen; Lp, lipoprotein; PG, proteoglycan; TC, total cholesterol; TIA, transient ischemic attack; Tx, transplantation

Normally distributed continuous variables are depicted as the mean ± standard deviation, continuous variables with a skewed distribution are given as the median [25th–75th percentile], and categorical variables are summarized by absolute numbers (percentages). Differences between tertiles of lipoprotein-proteoglycan binding susceptibility were tested using one-way ANOVA for normally distributed continuous variables, Kruskal-Wallis test for continuous variables with a skewed distribution, and Pearson's chi-squared test for categorical variables.

Subsequently, backward multiple linear regression analysis was used to assess which variables are determinants of LPBS in RTRs (Table 2). The concentration of TC (standardized β = −0.24, *P* < 0.001) and apoA-I (standardized β = −0.16, *P* < 0.001) was inversely associated with LPBS. Model *R*^2^ was 0.10.

**Table 2:** Predictors of lipoprotein binding susceptibility

	β	95% CI	Standardized β	*P*
Total cholesterol	−0.94	−0.12, −0.06	−0.24	<0.001
apoA-I	−0.24	−0.35, −0.12	−0.16	<0.001

All variables with *P* < 0.1 between tertiles were entered into a stepwise linear regression with backward elimination.

To further explore factors associated with LPBS, we first correlated the LDL-C/apoB ratio as an, allowedly, relatively crude but easy to calculate measure of the LDL size with LPBS. Previously, a smaller size of LDL particles had been identified as a determinant of increased binding to proteoglycans (23). Surprisingly, an overall significant positive correlation was observed in the RTR (r = 0.159, *P* < 0.001). To gain more insight, we used NMR lipoprotein particle number and sizing data that were available for n = 158 participants in our study. Interestingly, only small LDL had a positive correlation with LPBS (r = 0.20, *P* = 0.01), whereas medium- (r = −0.07, not significant) or large-sized LDL particles (r = 0.03, *P* = not significant) did not correlate with LPBS. Because oxidative modification had been shown to decrease binding of apoB-containing lipoproteins to proteoglycans (24), we measured in a subset of our cohort (n = 40) the conjugated diene content in these particles and correlated the result with LPBS. Consistent with previous reports, also in RTRs, a significant negative correlation between conjugated dienes and LPBS was observed (r = −0.46, *P* = 0.003). In addition, enzymatic modification by sPLA_2_-IIA was reported to increase binding of LDL to proteoglycans (23). Also, this result could be replicated in our cohort because plasma levels of sPLA_2_-IIA correlated positively with LPBS (r = 0.42, *P* = 0.016, n = 40). Because it has been previously demonstrated that Lp(a) is increased among patients with a low eGFR, (25) which in turn leads to a higher binding affinity to proteoglycans (26), we measured Lp(a) levels in our study population. Lp(a) was not correlated with LPBS (r = −0.06, *P* = 0.13). Furthermore, an adjusted Cox regression with Lp(a) as independent variable showed that there is no significant association between Lp(a) levels and GF (HR = 1.11, *P* = 0.25).

Data concerning specific causes of death were available for a median follow-up of 7 years. Of 130 (22%) patients who died in this period, 68 did so because of confirmed cardiovascular causes (12% of the total study population, 52% of the deceased patients). Furthermore, 29 (5% of the total study population, 22% of the deceased patients) patients died from malignancy, 23 (4% of the total study population, 18% of the deceased patients) from an infectious death, and 10 (2% of the total study population, 7% of the deceased patients) from other causes. During the median follow-up of 9.5 years for GF, a total of 73 (13%) subjects experienced this end point.

At baseline, LDL-C as well as LPBS were comparable between survivors and deceased RTRs, with respect to cardiovascular mortality (LDL-C: 3.90 ± 1.0 vs. 3.88 ± 1.0 mmol/l, *P* = 0.87; LPBS: 1.34 ± 0.42 vs. 1.29 ± 0.5 nmol/mmol, *P* = 0.33). While baseline LDL-C levels were also similar in patients developing GF or not (4.03 ± 1.38 vs. 3.88 ± 0.91 mmol/l, *P* = 0.22), LPBS was significantly higher in patients who subsequently developed GF than in those with a surviving graft (1.47 ± 0.63 vs. 1.32 ± 0.39 nmol/mmol, *P* = 0.003).

Cox regression analysis revealed that neither LPBS nor the classical biomarker LDL-C was associated with CVD mortality (Table 3); this conclusion remained valid after adjustment for a number of potential confounding factors in different statistical models. However, Cox regression analysis showed a prospective association between LPBS and chronic GF (HR, 1.87; 95% CI, 1.24–2.84; *P* = 0.003, Table 4, model 1). Adjusting for age and sex did not considerably reduce this association (HR, 1.84; 95% CI, 1.21–2.81; *P* = 0.004, Table 4, model 2).

**Table 3:** Comparison between the association of either LDL function (LPBS) or mass levels of LDL-C with cardiovascular mortality

	LPBS		LDL-C Concentration	
	HR [95% CI]	*P*	HR [95% CI]	*P*
Model 1	0.70 [0.38–1.30]	0.26	0.97 [0.75–1.25]	0.81
Model 2	0.74 [0.41–1.34]	0.32	0.98 [0.76–1.27]	0.89
Model 3	0.76 [0.41–1.39]	0.37	1.03 [0.80–1.32]	0.82
Model 4	0.74 [0.41–1.34]	0.32	0.97 [0.75–1.25]	0.80
Model 5	0.76 [0.42–1.38]	0.36	1.00 [0.77–1.29]	0.98
Model 6	0.76 [0.41–1.37]	0.36	1.00 [0.78–1.30]	0.97
Model 7	0.73 [0.40–1.35]	0.33	0.99 [0.76–1.28]	0.92
Model 8	0.72 [0.40–1.32]	0.29	0.99 [0.76–1.28]	0.93

HR, hazard ratio; LPBS, lipoprotein-proteoglycan binding susceptibility.

Model 1: crude; model 2: adjusted for age and sex; model 3: model 2+ adjustment diabetes mellitus; model 4: model 2+ adjustment for BMI; model 5: model 2+ adjustment for dialysis time and time between transplantation and inclusion; model 6: model 2+ adjustment for type of transplantation and donor age; model 7: model 2+ adjustment for use of calcineurin inhibitors and proliferation inhibitors; model 8: model 2+ adjustment for use of statins.

**Table 4:** Comparison between the association of either LDL function (LPBS) or mass levels of LDL-C with chronic graft failure

	LPBS		LDL-C Concentration	
	HR [95% CI]	*P*	HR [95% CI]	*P*
Model 1	1.87 [1.24–2.84]	0.003	1.13 [0.91–1.41]	0.25
Model 2	1.84 [1.21–2.81]	0.004	1.13 [0.91–1.41]	0.26
Model 3	1.85 [1.22–2.83]	0.003	1.15 [0.93–1.42]	0.20
Model 4	1.25 [0.85–1.82]	0.25	0.97 [0.78–1.20]	0.76
Model 5	1.85 [1.22–2.81]	0.004	1.14 [0.91–1.43]	0.24
Model 6	1.86 [1.23–2.83]	0.003	1.13 [0.90–1.42]	0.28
Model 7	1.89 [1.24–2.89]	0.003	1.13 [0.90–1.41]	0.29
Model 8	1.75 [1.15–2.69]	0.010	1.14 [0.90–1.45]	0.27

HR, hazard ratio; LPBS, lipoprotein-proteoglycan binding susceptibility.

Model 1: crude; model 2: adjusted for age and sex; model 3: model 2 + adjustment for use of statins; model 4: model 2 + adjustment for estimated glomerular filtration rate; model 5: model 2 + adjustment for period of acute rejection; model 6: model 2 + adjustment for number of human leukocyte antigen mismatches, primary renal disease and period of acute rejection; model 7: model 2+ adjustment for dialysis time and time between transplantation and baseline; model 8: model 2+ adjustment for type of transplantation and donor age.

Comparably, adjustment for a number of other potentially impacting factors, namely the use of statins, periods of acute rejection, number of HLA mismatches, primary renal disease, dialysis time, time between transplantation and inclusion, type of transplantation, and donor age did not appreciatively change the significance of the prospective association. However, after additional adjustments for eGFR, significance was lost (HR, 1.25, 95% CI, 0.85–1.82; *P* = 0.25, Table 4, model 7). We attempted to further delineate the relationship of LPBS and eGFR. Pearson’s correlation coefficients showed that there is no significant correlation between eGFR and LPBS (r = −0.03, *P* = 0.52). Then, an interaction term was computed and the association with GF was assessed (HR = 0.99, *P* = 0.40). This showed that there is no significant interaction between eGFR and LPBS.

In contrast to these findings with respect to the functional read-out of LPBS, LDL-C levels were not associated with GF, neither in univariate nor in all computed multivariable Cox regression models (Table 4).

Cox regression analyses were repeated with crude proteoglycan binding. The results were not substantially different with regard to the normalized marker of LPBS (supplemental Tables S1 and S2).

**Fig. 1:**
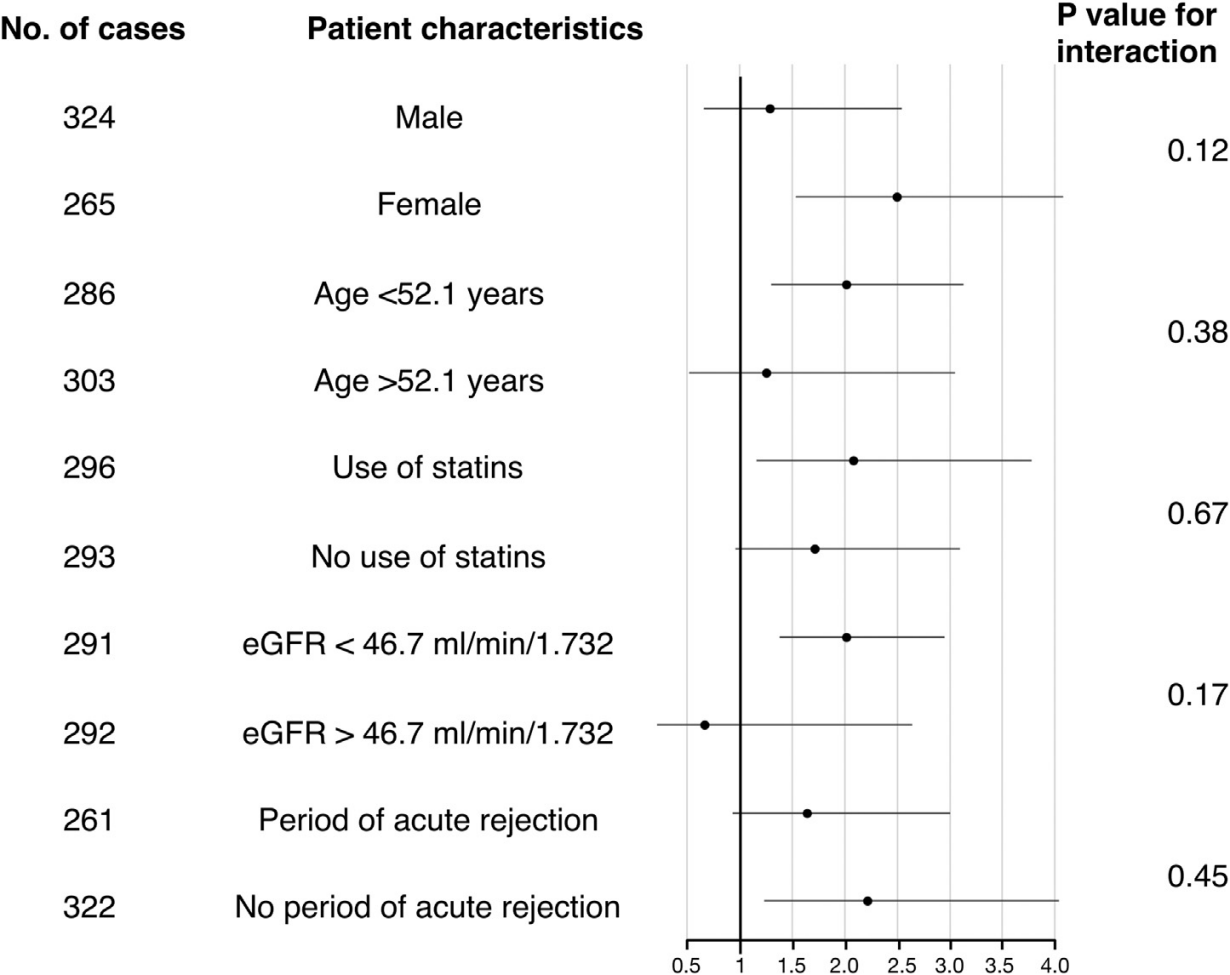
Hazard ratios for the association of LDL function (LPBS) with incident graft failure, by several participant level characteristics. eGFR, estimated glomerular filtration rate; LPBS, lipoprotein-proteoglycan binding susceptibility.

As shown in Fig. 1, the association of LPBS with chronic GF was not different for males versus females (*P* for interaction = 0.12), subjects with high versus low age (*P* for interaction = 0.38), use of statins versus no use of statins (*P* for interaction = 0.67), high versus low eGFR (*P* for interaction = 0.17), or period of acute rejection versus no period of acute rejection (*P* for interaction = 0.45). However, for the association of LPBS with CVD mortality, there was an interaction with the use of calcineurin inhibitors versus no use of calcineurin inhibitors (*P* for interaction = 0.03), indicating that the relationship of LPBS with CVD risk is stronger in subjects that use calcineurin inhibitors.

## Discussion

The results of this study demonstrate that beyond the static measurement of circulating LDL-C levels, functional metrics determining lipoprotein retention to proteoglycans such as LPBS can provide useful clinical information. Specifically, we show that in RTRs, LPBS was prospectively associated with chronic GF, a manifestation of de novo atherosclerosis, but not with CVD death, largely a consequence of preexisting, complex atherosclerotic lesions. We interpret these findings as being consistent with the response-to-retention hypothesis of atherosclerotic lesion formation (10). In contrast, LDL-C was neither associated with incident CVD mortality nor with chronic GF.

Although RTRs have a substantially elevated risk for atherosclerosis-related disease, classical biomarkers such as LDL-C or HDL-C levels fail to serve as predictors (1, 3, 5). Thus, the identification of patients at risk to develop either CVD events or accelerated chronic GF represents an unmet clinical need. Dynamic functional tests for proatherogenic properties of apoB-containing lipoproteins might offer a realistic chance to fill this current gap. Mechanistically, RTRs have an increased proinflammatory and oxidative stress load (27, 28, 29) that could conceivably contribute to modify apoB-containing lipoproteins in a way that they bind with higher affinity to vascular proteoglycans. However, the precise molecular mechanisms of this deserves further research. Although we consistently observed that a high LPBS is associated with measures of small LDL particles (NMR as well as LDL-C/apoB ratio), high LPBS was also associated with lower triglycerides. Given the inverse correlation between triglycerides and small LDL in the general population, especially the determinants of triglyceride metabolism in the setting of RTRs need to be better understood because this specific patient group potentially experiences the combined impact of kidney (dys)function, pre-existing disturbances in metabolism such as insulin resistance and immunosuppressive medication. It also appears relevant to include in such studies measures of oxidation in lipoproteins of different sizes because small LDLs bind better to proteoglycans but are on the other hand also more susceptible to oxidative modification (30), which would inhibit such binding (24). A multimodal approach covering a multitude of different potentially impacting factors could possibly help elucidate the perceived complex interaction of different processes and modifications relevant for the binding of lipoproteins to proteoglycans in RTRs. Conversely, and not addressed in the present work, the function and/or quantity (and hence atherogenicity) of proteoglycans could be altered as well in RTRs. Also here, important impacting factors are inflammation and oxidative stress. Oxidized LDL particles stimulate the production of modified proteoglycans with elongated chains, which consequently have a higher ability to retain LDL (31, 32). It is conceivable that a multiplicative effect of modifications in both lipoproteins and proteoglycans will lead to an even further enhanced binding susceptibility and subsequently increased atherosclerotic lesion formation. Research in rats found that expression of perlecan, a basement membrane-type heparan sulfate proteoglycan, is significantly increased in renal allografts after experimental transplantation, compared with nontransplanted control kidneys and isografts (33). The proteoglycan expression correlated with the severity of tissue remodeling and impaired graft function (33). This underlines, although challenging in clinical routine, the potential benefits of including proteoglycans present in the allograft’s extracellular matrix in individual TV risk analyses.

Potential therapeutic intervention options to decrease the interaction between LDL and proteoglycans are thus far limited. In vitro, glucosamine has been indicated to result in the production of proteoglycans by smooth muscle cells with a reduced binding capacity for LDL (34). However, dietary glucosamine supplementation resulted in increased atherosclerotic lesion formation in preclinical models limiting the applicability of such an approach (35, 36). Statins were shown to have a dual effect, decreasing LDL-proteoglycan binding (21), as well as the production of proteoglycans with reduced affinity to LDL (37). However, in our patients, statin use was not associated with any change in LPBS. Finally, measures to enrich LDL in cholesteryl oleate such as canola oil appear to reduce the binding of LDL to proteoglycans (38), but prospective clinical studies addressing this concept are not available.

A potential limitation of the current work is that this study is from a single center in the North of the Netherlands, thus representing a population with a relatively homogenous and also narrow Caucasian genetic background. Further replication would be required to inform, if our results are generalizable. In addition, although TransplantLines is one of the largest prospective renal transplantation cohorts, the number of participants is still limited, thus impacting predictive power. Future longitudinal research also needs to address if and in which direction the relationship of LPBS with graft function is causative. Next to our assumption that increased LPBS worsens graft function, it could mechanistically also be envisioned that a worsening graft function results in increased LDL modifications and thereby more LDL-proteoglycan binding (39). After all, a decreased eGFR is to date the strongest predictor for incident GF (40). To gain more mechanistic insights and distinguish lipid-driven from immune-mediated events (or elucidate an interdependency of these), it would be valuable to carry out serial histological evaluations of the vasculature of kidney grafts; however, due to the invasive nature of such work, respective studies are only possible to be carried out in preclinical models. Furthermore, although EDTA (a known antioxidant in addition to being an anticoagulant) plasma was used, sample generation and storage were similar for all patients and the assay was performed in an identical fashion for all samples using internal controls, a certain degree of oxidation cannot be formally excluded. In addition, despite differences in storage time being minor as opposed to time until the assay was performed, still samples were stored for a large number of years before analysis and different storage length of the samples might have a potential effect. Furthermore, the clinical implementation of LPBS as a biomarker is at this point difficult due to logistic challenges and lack of standardization; overcoming these represents a future challenge. With respect to the chosen assay setup, we would like to point out that, although the use of isolated LDL in proteoglycan binding studies revealed important pathophysiological information (10, 22, 39), the aim of our present work was to set up an assay that can be performed in large cohorts and, if proven useful, be further developed for routine clinical chemistry laboratories. However, feasibility challenges of routine LPBS studies still include a lack of availability of sufficient amounts of standardized arterial proteoglycans, as well as using isolated LDL in the setting of a clinical test. To address the latter issue, we also used human plasma per unit volume. This procedure has the advantage that no previous information regarding the samples is required and no isolation of a specific class of lipoproteins needs to be carried out that could potentially also impact lipoprotein composition or function. Importantly, another advantage of the chosen setup is that any potential factors in plasma, which could influence the interaction of LDL with proteoglycans, are still present.

In summary, the present study indicates that LPBS as a dynamic test for the individual proatherogenicity of LDL particles is associated with incident GF. This association was particularly pronounced in patients with a low eGFR. Our work suggests that focusing on the interaction of lipoproteins with extracellular matrix components could lead to the identification not only of useful personalized predictive biomarkers but also of potential pharmacological intervention targets. Thereby, unmet clinical needs in the patient population of RTR could be successfully addressed. The goal would be to reduce lipid deposition in the vascular wall of coronary arteries and kidney grafts to prevent chronic GF and possibly also CVD events.

## Data availability

The data that support the findings of this study are available from the corresponding author upon reasonable request.

## Supplemental data

This article contains supplemental data.

## Conflict of interest

M. A. C. is an employee of Labcorp. All other authors declare no conflicts of interest.
