# Supplementary material for: Proteoglycan binding as proatherogenic function metric of apoB-containing lipoproteins and chronic kidney graft failure
Source: J Lipid Res. 2021 Apr 30;62:100083. doi: 10.1016/j.jlr.2021.100083 (PMC8173310; doi:10.1016/j.jlr.2021.100083)
Supplement: Supplemental Table S1 and S2 [file mmc1.pdf]

Supplementary Online Content

Proteoglycan-binding as pro-atherogenic function metric of apoB-containing lipoproteins and chronic kidney graft failure

Hannah L.M. Steffen[1,*], Josephine L.C. Anderson[1,*], Margot L. Poot[1], Yu Lei[2], Margery A. Connelly[3], Stephan J.L. Bakker[4], Katariina Öörni[5,6,*], Uwe J.F. Tietge[1,2,7,*,#]

[1] Department of Pediatrics, [4] Department of Nephrology, University of Groningen, University Medical Center Groningen, 9713 GZ Groningen, The Netherlands

[2] Division of Clinical Chemistry, Department of Laboratory Medicine, Karolinska Institutet, Stockholm, Sweden

[3] Laboratory Corporation of America Holdings (LabCorp), Morrisville, NC 27560, USA

[5] Atherosclerosis Research Laboratory, Wihuri Research Institute, Helsinki, Finland

[6] Molecular and Integrative Bioscience Research Programme, Faculty of Biological and Environmental Sciences, University of Helsinki, Helsinki, Finland

[7] Clinical Chemistry, Karolinska University Laboratory, Karolinska University Hospital, Stockholm, Sweden

*These authors contributed equally to this work

Supplemental Table S1. The association of crude LPBS with cardiovascular disease mortality

	**HR [95% CI]**	***p***
Model 1	0.91 [0.79- 1.04]	0.18
Model 2	0.93 [0.81- 1.06]	0.28
Model 3	0.94 [0.82- 1.08]	0.40
Model 4	0.93 [0.81- 1.06]	0.28
Model 5	0.93 [0.81- 1.06]	0.29
Model 6	0.93 [0.81- 1.06]	0.26
Model 7	0.92 [0.81- 1.07]	0.29
Model 8	0.95 [0.83- 1.09]	0.46

Model 1: crude; model 2: adjusted for age and gender; model 3: model 2+ adjustment diabetes mellitus; model 4: model 2+ adjustment for body mass index; model 5: model 2+ adjustment for dialysis time and time between transplantation and inclusion; model 6: model 2+ adjustment for type of transplantation and donor age; model 7: model 2+ adjustment for use of calcineurin inhibitors and proliferation inhibitors; model 8: model 2+ adjustment for use of statins. Abbreviations: LPBS, lipoprotein-proteoglycan binding susceptibility; LDL-C, low-density lipoprotein; HR, hazard ratio; CI, confidence interval.

Supplemental Table S2. The association of crude LPBS with chronic graft failure

	**HR [95% CI]**	***p***
Model 1	1.14 [1.02- 1.27]	0.02
Model 2	1.14 [1.02- 1.27]	0.03
Model 3	1.14 [1.02- 1.28]	0.02
Model 4	1.02 [0.92- 1.14]	0.68
Model 5	1.14 [1.02- 1.27]	0.02
Model 6	1.14 [1.02- 1.27]	0.02
Model 7	1.14 [1.02- 1.28]	0.02
Model 8	1.12 [1.00- 1.25]	0.055

Model 1: crude; model 2: adjusted for age and gender; model 3: model 2 + adjustment for use of statins; model 4: model 2 + adjustment for estimated glomerular filtration rate; model 5: model 2 + adjustment for period of acute rejection; model 6: model 2 + adjustment for number of human leukocyte antigen mismatches, primary renal disease and period of acute rejection; model 7: model 2+ adjustment for dialysis time and time between transplantation and baseline; model 8: model 2+ adjustment for type of transplantation and donor age. (Abbreviations: LPBS, lipoprotein-proteoglycan binding susceptibility; LDL-C, low-density lipoprotein; HR, hazard ratio; CI, confidence interval.)
